# An unusual case of collagenous gastritis in a middle- aged woman with systemic lupus erythromatosis: a case report

**DOI:** 10.1186/1752-1947-8-278

**Published:** 2014-08-18

**Authors:** Asma Al-Kandari, Hossam Al-Alardati, Hassan Sayadi, Bandar Al-Judaibi, Mohammad Mawardi

**Affiliations:** 1 King Faisal Specialist Hospital & Research Centre, Al Rawdah, Jeddah 23433, Saudi Arabia; 2 Department of Medicine, Division of Gastroenterology, London Health Science Center, The University of Western Ontario, 339 Windermere Road, London, ON N6G 2V4, Canada; 3 Department of Medicine, Division of Gastroenterology, King Saud University, Riyadh 12746, Saudi Arabia; 4 Multi-Organ Transplant Program, Western University, 339 Windermere Road, London, ON N6G 2V4, Canada

**Keywords:** Collagenous gastritis, Collagen band

## Abstract

**Introduction:**

Collagenous gastritis is a rare histopathologic disease. It is characterized by marked subepithelial collagen deposition with associated inflammatory infiltrate. It is considered an uncommon disease among the general population. Collagenous gastritis without colonic involvement is an extremely rare disease. It is not known to be associated with systemic lupus erythromatosis. This is the first report of this type of association.

**Case presentation:**

We present a 47-year-old woman from southeast Asia with dyspepsia and mild anemia. Her past medical history was significant for systemic lupus erythromatosis, autoimmune hemolytic anemia as well as hypothyroidism. Her gastroscopy and colonoscopy results were normal from an endoscopic point of view. However, the histopathology showed collagenous gastritis.

**Conclusions:**

To the best of our knowledge, this is the first case reported of a patient with systemic lupus erythromatosis associated with collagenous gastritis. Further studies are needed to evaluate the association between both diseases from a pathophysiological and immunological perspective.

## Introduction

Collagenous gastritis is considered an uncommon disease among the general population. It was discovered first by Colletti and Trainer in 1989 [1]. It is characterized by thickening of the collagen band under the mucosal epithelium by more than 10μm and infiltration of inflammatory cells in the lamina propria of the mucous membrane, similar to collagenous colitis [2]. The etiology of this disease is still unclear. However, constant signs of immune activation, including overexpression of HLA-DR by epithelial cells and CD25-positive cells in the lamina propria, are seen in the gastric biopsy from patients with collagenous gastritis; these activated immune cells produce cytokines and growth factors that could stimulate the production of extracellular matrix leading to collagenous deposition [3]. It is not gender specific. Collagenous gastroduodenitis without colonic involvement is exceptionally rare [4]. Patients usually present with severe anemia. Most of them have been associated with colonic involvement, the clinical picture is of predominately chronic watery diarrhea and weight loss. The clinical presentation depends on the region of the gastrointestinal tract involved. Collagenous gastroduodenitis usually presents with chronic watery diarrhea and weight loss most likely due to the association with collagenous colitis.

## Case presentation

We report a 47-year-old woman of southeast Asian ethnicity who presented to the clinic with a two-month history of epigastric pain. Our patient described the pain as a dull ache in nature, worsened by ingestion of cold drinks and associated with reflux symptoms. She denied having any nausea, vomiting, diarrhea, gastrointestinal hemorrhage or fever, and she claimed that her appetite was normal. Her past medical history included systemic lupus erythromatosis, autoimmune hemolytic anemia, and hypothyroidism, and she was being maintained on chloroquine and a low dose of corticosteroids. Both diseases were diagnosed seven years ago.

**Figure 1:**
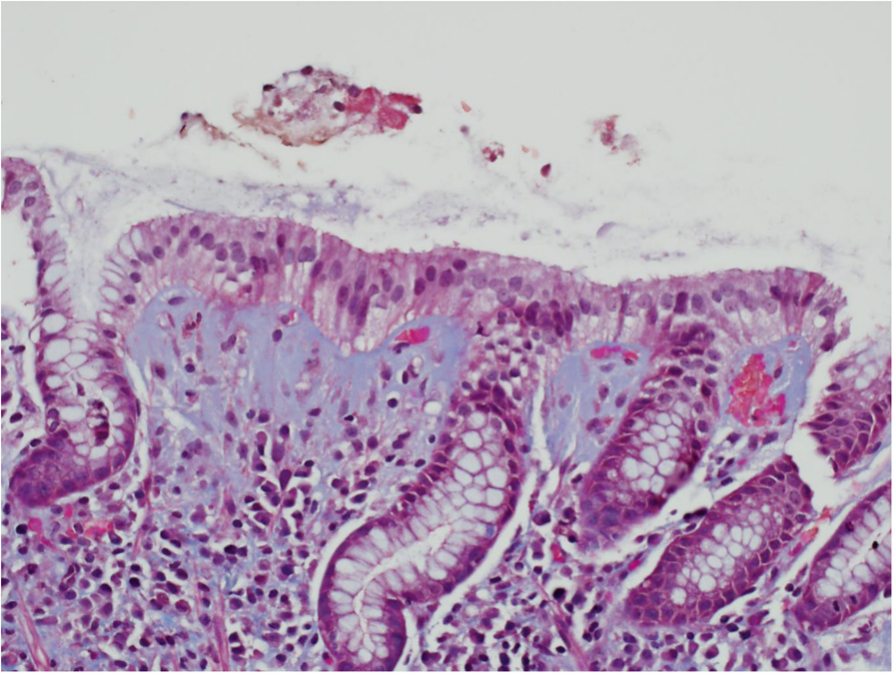
**Gastric biopsy showing the blue-stained subepithelial collagenous layer by trichrome stain.** (Trichrome; ×400 magnification).

**Figure 2:**
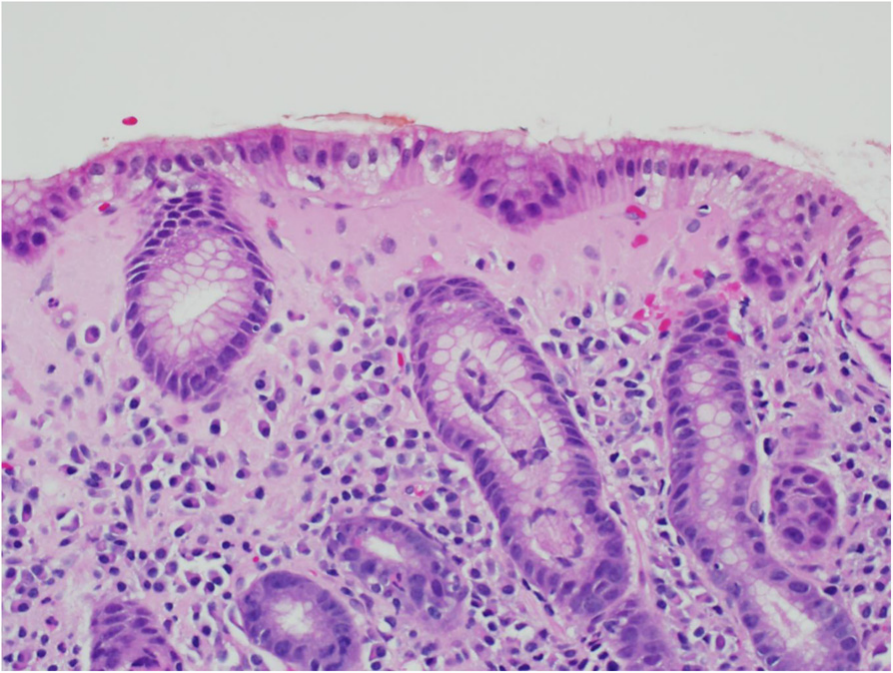
**The subepithelial layer is thickened with a homogenous, lightly eosinophilic collagenous layer.** There is associated lymphoplasmacytic inflammation in the lamina propria. There is focal acute inflammation involving the gland crypts. (Note the crypt at the central lower half of the figure). (Hematoxylin and eosin (H&E) stain; ×400 magnification).

On examination, she had mild epigastric tenderness. Her laboratory investigations revealed a hemoglobin level of 101g/L (normal 115 to 160), and a mean corpuscular volume (MCV) of 96.7 (normal 79 to 97). The rest of the initial investigation results, including albumin, vitamin B12 and folate tests, were normal. Due to her significant anemia and severe epigastric pain, she underwent esophagogastroduodenoscopy and colonoscopy. The colonoscopy, which including random colonic biopsies, was normal. Likewise, the esophagogastroduodenoscopy was normal. However, gastric biopsies showed a thickened subepithelial layer with associated gastritis. The subepithelial collagenous layer was homogenous, lightly eosinophilic on hematoxylin and eosin (H&E), and stained with trichrome stain (Figure 1). There were few entrapped fibroblasts, inflammatory cells and blood vessels in the collagenous layer. It was approximately 70μm thick. The inflammatory infiltrate was predominantly lymphoplasmacytic with few neutrophils and eosinophils. Focal active inflammation in the form of cryptitis and reactive glands was present (Figure 2). The test for *Helicobacter pylori* was positive. She was started on a proton pump inhibitor (PPI) and 10 days of *H. pylori* eradication therapy. Over the next three months of follow-up, our patient’s symptoms improved.

## Discussion

This is the first case to be reported of collagenous gastritis with an underlying history of systemic lupus erythromatosis. The patient’s clinical presentation was probably consistent with type 1 disease with anemia but with no associated colonic involvement, although at her age, it is more common to have type 2 disease with an associated collagenous colitis. Due to the frequent association with immune-related disorders, including celiac disease, collagenous colitis, inflammatory bowel disease and a variety of systemic autoimmune disease, collectively these associations support the hypothesis that collagenous gastroenteritides have an immune activation, including the overexpression of HLA-DR by epithelial cells and CD25-positive cells in the lamina propria seen in the gastric biopsy [5]. Those activated immune cells produce cytokines and growth factors that stimulate the production of the extracellular matrix leading to collagen deposition. Collagenous gastritis usually presents with anemia from upper gastrointestinal bleeding and epigastric pain [6]. On endoscopy, the involved mucosa appears thickened and nodular with a diffuse cobblestone appearance. In the stomach the nodularity mainly involves the gastric body, but it is not seen in all cases [7]. Other reported findings include gastric mucosal erythema, erosions, hemorrhages, ulcerations and exudates [1]. The diagnosis of collagenous gastritis is made by histology, which shows distinctive findings. The etiology of collagenous gastritis is unclear. In general, it is considered a chronic persistent histologic disease characterized by a chronic intermittent clinical course in the majority of adult patients [8]. There is no significant mortality risk or periods of severe deterioration [8]. Collagen thickness has been associated with disease duration but not with disease severity. There have been no reports of carcinoma, lymphoma, or definitive dysplasia developing in association with collagenous gastritis [9]. There are no established treatment protocols for collagenous gastroenteritides, including collagenous gastritis, and resolution of the abnormalities either endoscopic or histologic has not been documented. Various therapies have been tried for collagenous gastritis including corticosteroids, ranitidine, omeprazole, misoprostol, sucralfate, aminosalicylates, sulfasalazine, cholysteramine and a hypoallergenic diet with marginal results [2,6,7,10].

## Conclusions

To the best of our knowledge, this is the first case to be reported of collagenous gastritis with an underlying history of systemic lupus erythromatosis. Gastroenterologists and pathologists needed to be aware of this condition when evaluating patients with epigastric pain, anemia, and dyspepsia. To the best of our knowledge, there is no literature evidence of an association of collagenous gastritis with systemic lupus erythromatosis.

## Consent

Written informed consent was obtained from the patient for publication of this case report and any accompanying images. A copy of the written consent is available for review by the Editor-in-Chief of this journal.

## Competing interests

The authors declare that they have no competing interests.

## Authors’ contributions

AA analyzed and interpreted the patient data regarding the collagenous gastritis and systemic lupus erythromatosis. HA and HS helped in the interpretation of the pathology of the gastric and colonic biopsy in the diagnosis of collagenous gastritis. BA assisted with the literature review assessment. MM supervised and assisted with the literature review and gathering the whole information for publication. All authors read and approved the final manuscript.
